# mtDNA heteroplasmy gives rise to a new maternal lineage in North Pacific humpback whales (*Megaptera novaeangliae*)

**DOI:** 10.1093/jhered/esac042

**Published:** 2022-09-23

**Authors:** Sophie P Pierszalowski, Debbie J Steel, Christine M Gabriele, Janet L Neilson, Phoebe B S Vanselow, Jennifer A Cedarleaf, Janice M Straley, C Scott Baker

**Affiliations:** Marine Mammal Institute and Department of Fisheries, Wildlife and Conservation Sciences, Oregon State University, Hatfield Marine Science Center, Newport, OR, USA; Marine Mammal Institute and Department of Fisheries, Wildlife and Conservation Sciences, Oregon State University, Hatfield Marine Science Center, Newport, OR, USA; Resource Management, Glacier Bay National Park and Preserve, Gustavus, AK, USA; Resource Management, Glacier Bay National Park and Preserve, Gustavus, AK, USA; Resource Management, Glacier Bay National Park and Preserve, Gustavus, AK, USA; College of Natural Resources, University of Alaska Southeast Sitka Campus, Sitka, AK, USA; College of Natural Resources, University of Alaska Southeast Sitka Campus, Sitka, AK, USA; Marine Mammal Institute and Department of Fisheries, Wildlife and Conservation Sciences, Oregon State University, Hatfield Marine Science Center, Newport, OR, USA

**Keywords:** germ-line fixation, haplotype, inheritance, mitochondrial DNA, regional fidelity, segregation

## Abstract

Heteroplasmy in the mitochondrial genome offers a rare opportunity to track the evolution of a newly arising maternal lineage in populations of non-model species. Here, we identified a previously unreported mitochondrial DNA haplotype while assembling an integrated database of DNA profiles and photo-identification records from humpback whales in southeastern Alaska (SEAK). The haplotype, referred to as *A8*, was shared by only 2 individuals, a mature female with her female calf, and differed by only a single base pair from a common haplotype in the North Pacific, referred to as *A−*. To investigate the origins of the *A8* haplotype, we reviewed *n* = 1,089 electropherograms (including replicate samples) of *n* = 710 individuals with *A−* haplotypes from an existing collection. From this review, we found 20 individuals with clear evidence of heteroplasmy for *A−*/*A8* (parental/derived) haplotypes. Of these, 15 were encountered in SEAK, 4 were encountered on the Hawaiian breeding ground (the primary migratory destination for whales in SEAK), and 1 was encountered in the northern Gulf of Alaska. We used genotype exclusion and likelihood to identify one of the heteroplasmic females as the likely mother of the *A8* cow and grandmother of the *A8* calf, establishing the inheritance and germ-line fixation of the new haplotype from the parental heteroplasmy. The mutation leading to this heteroplasmy and the fixation of the *A8* haplotype provide an opportunity to document the population dynamics and regional fidelity of a newly arising maternal lineage in a population recovering from exploitation.

## Introduction

A new mitochondrial DNA (mtDNA) haplotype can reach fixation in the female germ-line (hereafter, referred to simply as fixation) through heteroplasmy, i.e., the presence of more than one variant of the mitochondrial genome within a single individual (Hoeh et al. 1991). Heteroplasmy can originate from either length variants or point mutations, resulting in individuals with both derived and ancestral (or parental) haplotypes (Mignotte et al. 1990). Although heteroplasmy can also arise via mutation within the somatic tissue of an individual, only heteroplasmy in the female germ-line is inherited in subsequent generations (Birky 1978). During oogenesis or embryogenesis, there is considerable fluctuation in the number of mitochondrial organelles per cell, causing only a portion of the mtDNA to proliferate in successive generations (Bergstrom and Pritchard 1998). If heteroplasmy exists in a primordial germ cell or zygote of an adult female, this cellular bottleneck can generate the rapid segregation of haplotype variants leading to random genetic drift and possible fixation of either the parental or derived (i.e. mutant) haplotype in the mature oocyte or in the germ-line of the developing embryo (Chinnery et al. 2000). An alternative source of heteroplasmy, albeit rare in mammals, is paternal leakage, where mitochondria from the sperm enter the ova during fertilization and persist through embryogenesis (Wolff and Gemmell 2008).

mtDNA heteroplasmy has been reported in a number of animal species (e.g. Hoeh et al. 1991; Wilkinson and Chapman 1991; Steel et al. 2000; Kvist et al. 2003), including cetaceans (Vollmer et al. 2011), and specifically, the humpback whale (Baker et al. 1990, 2013; Larsen et al. 1996). Although several studies have reported rapid intergenerational fixation of heteroplasmic point mutations in domesticated mammal species (Ashley et al. 1989, Koehler et al. 1991), there is only one study that has documented the origins of a new haplotype through the segregation of a heteroplasmic lineage in a cetacean species. McLeod and White (2009) used a long-term dataset of photo-identification (ID) records and DNA profiles to trace the inheritance of heteroplasmy and fixation of a new haplotype through multiple generations of North Atlantic right whales (*Eubalaena glacialis*), a species with especially low mtDNA diversity.

Large-scale population surveys establish a baseline of existing genetic diversity and offer the potential to identify newly arising mtDNA haplotypes. Most relevant to this study, the *Structure of Populations, Levels of Abundance and Status of Humpback Whales* (SPLASH) project, was initiated to better understand the abundance and population structure of the North Pacific humpback whale (Calambokidis et al. 2008). During the SPLASH program (from 2004 to 2006), photo-identification records were collected and DNA profiles were generated for individuals from all known North Pacific humpback whale feeding and breeding grounds. From 1,803 individuals with mtDNA control region sequences, 28 mtDNA haplotypes were resolved, showing marked differences in frequencies among 10 recognized feeding regions (Baker et al. 2013). This regional differentiation of mtDNA haplotypes across North Pacific feeding grounds provides evidence that fidelity is maternally directed; a hypothesis first proposed based on high rates of return to specific feeding regions documented by photo-identification (Martin et al. 1984; Clapham and Mayo 1987, Baker et al. 1987).

In southeastern Alaska (SEAK), this large-scale population survey has been augmented by decades of dedicated research on humpback whales. This has generated a large collection of data on the distribution, seasonal return and life-history parameters (e.g. age and recorded mother/offspring relationships) of individual whales. These records demonstrate a notable degree of local site fidelity over extended periods, i.e., in some cases, up to 4 decades (Pierszalowski et al. 2016; Gabriele et al. 2017). Similar to right whales in the North Atlantic (McLeod and White 2009), humpback whales in SEAK show notably low haplotype diversity (Baker et al. 2013). During the SPLASH effort, only 6 haplotypes were found in a sample of 183 humpback whales from SEAK, with the large majority of individuals having either an *A+* (27%) or *A*− (66%) haplotype.

Here, we describe the origin of a new mtDNA haplotype in the North Pacific, first detected in SEAK, and the number and spatial distribution of individuals with heteroplasmy for the parental and the newly arising maternal lineage. The extensive photo-identification records and DNA profiles available for humpback whales in SEAK, together with the population’s low haplotype diversity, provided the opportunity to detect a point-mutation heteroplasmy that had been initially dismissed as a sequencing artifact. Results from this study support the value in maintaining long-term, individual-based records, with associated genetic samples for tracking a newly arising mutation and subsequent evolution within populations.

## Materials and methods

### Photo identification

Two related databases of photo-identification records were available for use in this study: 1) the SEAK Regional Database, with contributions from Glacier Bay National Park (GBNP) and the University of Alaska Southeast (UAS) and 2) the ocean-basin wide SPLASH database (Calambokidis et al. 2008). Photo-identification records of humpback whales have been collected in SEAK since the late 1960s by various research groups, including Sea Search, Ltd. (1968–1981), J. Straley Investigations and the UAS (1979–present), the University of Hawaii (1980–1984), the GBNP Humpback Whale Monitoring Program (1985–present), and as part of the ocean-basin study, SPLASH (2004–2005). Consistent stewardship of these data by GBNP and UAS has resulted in one of the longest-running photo-identification monitoring efforts of a whale species anywhere in the world (e.g., Gabriele et al. 2017). The SEAK Regional Database includes encounter histories for approximately 3,500 individual humpback whales in SEAK from 1973 to present. Individual whales that are sighted more than once were assigned a sequential southeastern Alaska identification code (SEAK ID). Many maternal relationships and ages inferred for individual whales from photo-identification observations of females with dependent calves are available within this collection (e.g. Gabriele et al. 2007; Pierszalowski et al. 2016; Neilson et al. 2018).

SPLASH photo-identification efforts extended to all known North Pacific humpback whale winter breeding grounds (in 2004, 2005, 2006) and all known summer feeding regions (in 2005, 2006). There were a total of 18,469 encounters with 7,971 individual humpback whales (Calambokidis et al. 2008). Here we refer primarily to a subset of SPLASH records with associated DNA profiles (Baker et al. 2013).

### Genetic samples and DNA profiling

Genetic samples utilized in this study were either 1) selected from a comprehensive collection of 1,026 SEAK tissue samples (692 with an associated SEAK ID), representing 25 years of genetic sampling in SEAK, or 2) selected from the ocean-basin wide SPLASH database. Most samples were collected using a small biopsy dart (Lambertsen 1987). DNA profiling included 500 bp of the mtDNA control region, 10 microsatellite loci and sex, following methods described previously for SPLASH (Baker et al. 2013).

### Identification of heteroplasmy and the derived haplotype

The mtDNA haplotypes from the SEAK Regional Database were compared to the 28 haplotypes previously characterized from DNA profiles of 1,803 individual whales in the SPLASH database (Baker et al. 2013). The collection of haplotypes from SPLASH represents approximately 8% of the estimated oceanic population of 21,808 humpback whales, at the time of the project (Barlow et al. 2011). Following the identification of a novel variant in the *A*− haplotypes, we reviewed all electropherograms of this common haplotype resulting from tissue samples collected in SEAK from 1987 to 2012. All *A*− and unspecified heteroplasmic sequences generated from samples collected throughout the North Pacific during the SPLASH project from 2004 to 2006 were also reviewed for heteroplasmy at this position in the control region. Heteroplasmy was initially assessed by setting the secondary peak-height filter at 20% in the program *Sequencher* (Gene Codes). This threshold was considered conservative given reports that experimental mixtures of 10% can be detected reliably with conventional capillary sequencing (Ramos et al. 2013). All electropherograms reporting a secondary peak height of >20% at the position of the new substitution were visually confirmed.

### Maternity analysis

To investigate the potential for inheritance and fixation of a new haplotype from the parental heteroplasmy, we conducted a maternity analysis in *CERVUS* v3.0 (Kalinowski et al. 2007). Likelihood values for each mother/offspring pair were generated in *CERVUS*. A female was considered a likely mother if she was assigned the highest, positive logarithm of the odds (LOD) score and shared an allele at 8 or more loci when compared with the putative offspring (hereafter referred to as “confirmed by genotyping”).

### Encounter locations

**Fig. 1.**
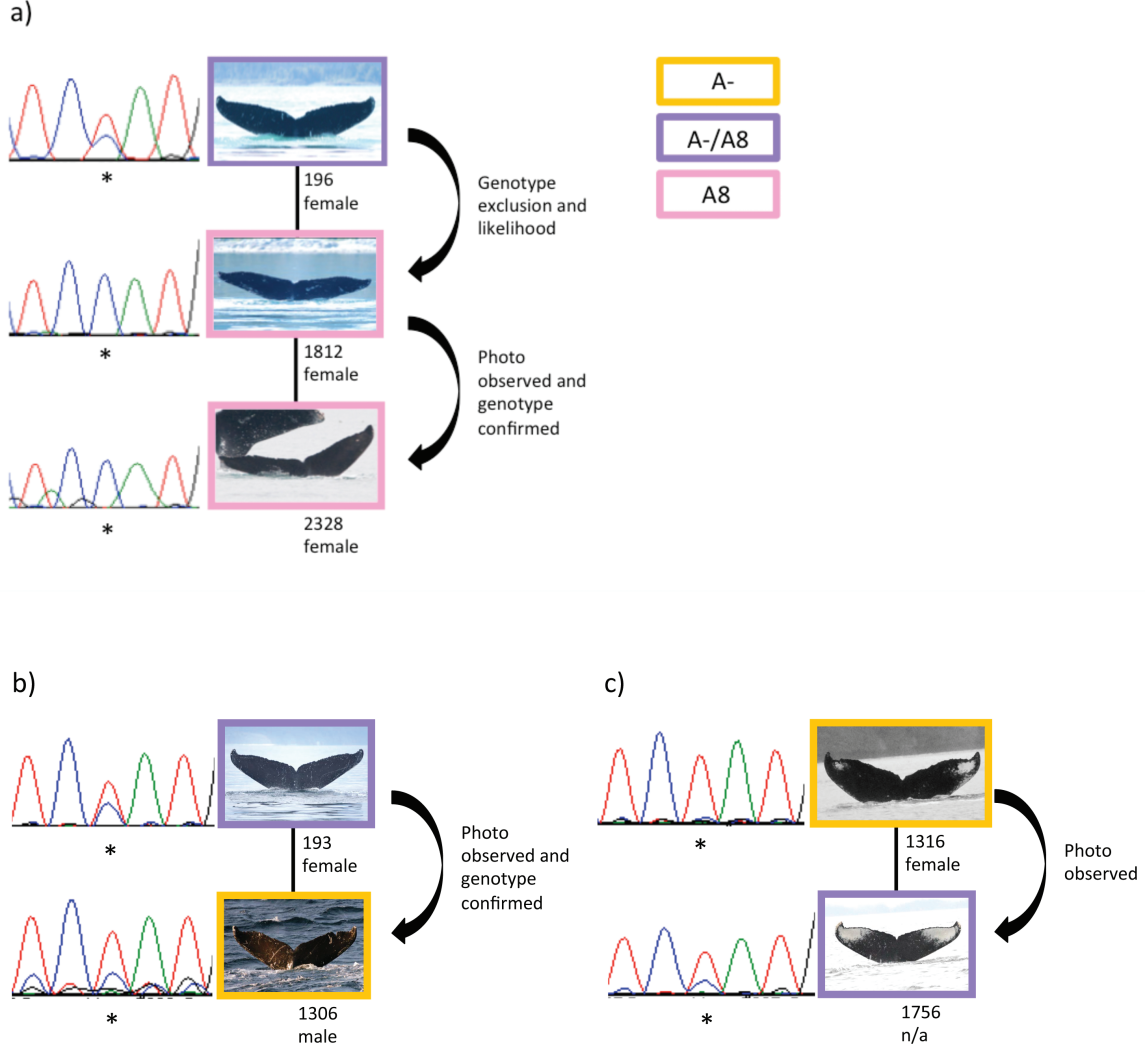
Alternate scenarios for inheritance of mtDNA heteroplasmy in humpback whales using confirmed mother/offspring relationships. (a) The pedigree of the 2 *A8* individuals and the most likely *A−*/*A8* heteroplasmic mother of SEAK ID 1812. (b and c) Alternate scenarios for inheritance of heteroplasmy for photo observed and photo observed/genotype confirmed relationships of *A−*/*A8* heteroplasmic North Pacific humpback whales with relatives that also had an available haplotype. SEAK ID and sex are shown below each fluke image. Electropherograms show relative peak height at position 283 (indicated with an asterisk) for heteroplasmic and homoplasmic individuals, showing cytosine as blue and thymine as red.

To investigate the spatial distribution of the newly arising lineage, we mapped all encounter locations of individuals using SPLASH records in *ArcGIS* 10.1 and highlighted encounters of individuals with the *A−/A8* heteroplasmy. The SPLASH database was used rather than the SEAK database because it characterized a more representative sampling effort across the North Pacific (Fig. 1). There was a total of 18,469 encounters with 7,971 individuals across the North Pacific in the SPLASH project (Calambokidis et al. 2008). Only encounters of heteroplasmic individuals with an existing DNA profile during SPLASH are highlighted on the map.

## Results

### Discovery of a new haplotype, A8

Review of the SEAK Regional Database revealed a new mtDNA haplotype, referred to here as *A8*, differing from the common *A−* haplotype by a transition from T to C at position 283 (Table 1). We identified 2 individuals with this novel substitution, both sampled in 2010 (after the SPLASH project). Review of photo-identification records, and confirmation with genotypes, revealed that the 2 *A8* individuals were an observed mother and her offspring, referred to as SEAK ID 1812 (female, first sighted in SEAK in 2003) and SEAK ID 2328 (female, born in 2010). There was no photo-identification record of an observed mother for SEAK ID 1812, the *A8* mother.

**Table 1.** Relative positions of variable nucleotides at sites defining the 6 mtDNA control region haplotypes identified in humpback whales collected in SEAK from 1987 to 2012, as archived within the SEAK DNA Registry.

Haplotype Code	Genbank #	Nucleotides at Variable Sites																
		23	82	83	98	123	131	143	159	243	262	264	266	270	283	313	314	489
*A+*	KF477244	G	T	T	C	C	G	T	T	T	C	A	T	A	T	T	C	A
*A4*	KF477247	□	C	□	□	□	□	□	□	□	□	□	□	□	□	□	□	□
*A−*	KF477245	A	□	□	□	□	□	□	□	□	□	□	□	□	□	□	□	□
*A8*	OP185707	A	□	□	□	□	□	□	□	□	□	□	□	□	C	□	□	□
*E2*	KF477256	□	□	□	□	□	□	□	□	□	□	G	C	□	□	□	□	□
*F2*	KF477266	□	□	C	A	T	A	C	C	C	T	□	□	G	□	C	T	G

Boxes (□) indicate matches with the reference sequence (*A+*).

### Identifying mtDNA heteroplasmy

To investigate the origin of the novel *A8* haplotype, we reviewed electropherograms of *n* = 355 individuals with *A−* haplotypes included in the SEAK database. This revealed *n* = 15 individuals that showed a secondary peak height of at least 20% for either the *A−* or *A8* haplotype at site 283. Outside of SEAK, review of electropherograms of *n* = 355 individuals with *A−* haplotypes or unspecified heteroplasmic sequences sampled in the North Pacific during SPLASH (2004–2006) revealed another *n* = 5 (Supplementary Material, Table 1). This heteroplasmy was consistent with a single base-pair change for nucleotides C and T at position 283 (haplotype *A−*/*A8*). It should be noted that other individuals had a minor secondary peak that did not meet the 20% secondary peak criteria; thus, the number of individuals with reported *A−*/*A8* heteroplasmy (*n* = 20) is likely to be an underestimate.

### Inheritance and segregation of A8

We searched for a potential heteroplasmic mother of SEAK ID 1812 (the *A8* cow) using genotype exclusion and likelihood. We included all confirmed *A−*/*A8* heteroplasmic females with an available genotype as well as *A−*/*A8* individuals with a genotype but unknown sex as putative mothers (*n* = 11, Table 2). SEAK ID 196 was the only candidate to share one allele at every locus with SEAK ID 1812 (i.e. the Mendelian expectation of a parent/offspring relationship, Supplementary Material, Table 2). This inference was supported by a high LOD score (6.23) and a low nonexclusion probability for SEAK ID 1812 (0.007). Based on this evidence, it is likely that the inheritance and segregation of the heteroplasmic haplotype and the subsequent fixation of the *A8* haplotype occurred between a heteroplasmic mother (SEAK ID 196, haplotype *A−*/*A8*) and her homoplasmic offspring (SEAK ID 1812, haplotype *A8*).

**Table 2.** Maternity analyses for the individual humpback whale, SEAK ID 1812 (the *A8* cow), using all *A−*/*A8* heteroplasmic females as candidate mothers.

Candidate mother’s SEAK ID/SPLASH ID	Loci compared	Loci mismatching	Pair LOD score
196/n.a.	9	0	6.23
215/474268	10	1	−0.30
1345/n.a.	10	1	−2.03
193/ 474448	9	2	−5.36
1795/574356	10	2	−5.62
n.a./700572	10	3	−10.13
n.a./430048	10	3	−10.19
2119/700630	10	4	−14.06
n.a./700227	10	4	−14.34
1336/474397	10	4	−15.55
n.a./430294	10	5	−19.19

The number of mismatching loci and the LOD score for pairwise comparisons of SEAK ID 1812 with each candidate mother. LOD scores were calculated in *CERVUS* v3.0 (table sorted by LOD score). The nonexclusion probability for SEAK ID 1812 was 0.007.

### Relatedness within a heteroplasmic lineage

To further investigate the inheritance of heteroplasmy, the SEAK Regional Database was reviewed for observed mother/offspring relationships involving *A−*/*A8* heteroplasmic and *A8* individuals. Three *A−*/*A8* heteroplasmic and *A8* individuals had offspring with a known haplotype. As noted above, SEAK ID 196 (*A−*/*A8*) is the most likely mother of SEAK ID 1812 (*A8*), who is the mother of SEAK ID 2328 (*A8*). The relationship between SEAK IDs 1812 and 2328 was observed with photo-identification and confirmed by genotyping. SEAK ID 193 (*A−*/*A8*) is the observed mother of SEAK ID 1306 (*A−*). The relationship between SEAK IDs 193 and 1306 was confirmed by genotyping. SEAK ID 1316 (*A−*) is the photo-identification observed mother of SEAK ID 1756 (*A−*/*A8*). A genotype was not available for SEAK ID 1756 to confirm the relationship. A pedigree representing *A−*/*A8* individuals and relatives with known haplotypes is shown in Fig. 1. This alternate fixation of parental and derived mtDNA haplotypes is likely caused by a fluctuation in the number of mitochondrial organelles per cell during embryogenesis or oogenesis.

### Regional fidelity of the heteroplasmic lineage

**Fig. 2.**
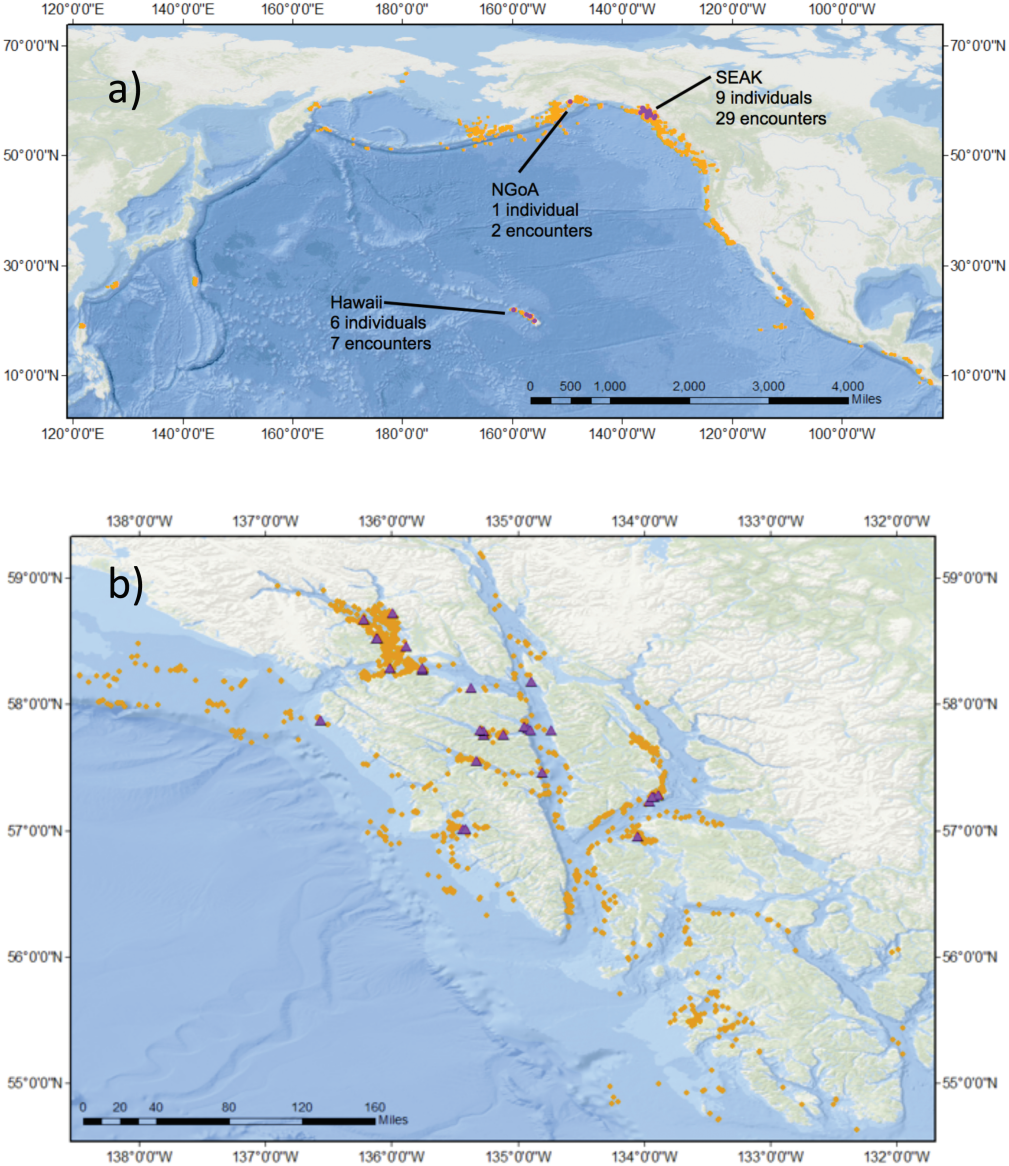
Distribution of all SPLASH encounters with humpback whales, showing sighting locations of individuals with the newly arising *A8 haplotypes or A−*/*A8* heteroplasmy (purple triangles) and all other individuals (orange diamonds). (a) The encounters across the North Pacific, including the Northern Gulf of Alaska (NGoA), and (b) Encounters within southeastern Alaska (SEAK). Note that 2 individuals were encountered in both SEAK and Hawaii. The SPLASH records represent 18,469 encounters with 7,971 individuals during the 3-year study, 2004–2006 (Calambokidis et al. 2008).

Encounter locations of the 14 individuals with *A−/A8* heteroplasmy that were sampled as part of the SPLASH project are shown highlighted among the 18,469 total encounters from SPLASH in Fig. 2. Seven individuals were encountered only within the SEAK feeding region (*n* = 18 encounters), 4 were encountered only on the Hawaiian breeding grounds (*n* = 5 encounters), 2 individuals were encountered in both SEAK (*n* = 11 encounters) and Hawaii (*n* = 2 encounters), and 1 was encountered only in the Northern Gulf of Alaska feeding region (*n* = 2 encounters).

The 6 *A−*/*A8* heteroplasmic individuals identified in this study that were not sampled during the SPLASH project were all sampled in SEAK. Both *A8* individuals were encountered in SEAK in 2010 and have only been encountered there; neither of these were sampled for DNA profiling during SPLASH.

## Discussion

### Discovery of a New North Pacific haplotype and heteroplasmic origins

We provide the first documentation of a newly arising haplotype among humpback whales in the North Pacific. This haplotype was not reported in the previous SPLASH project, despite extensive sampling coverage across the entire North Pacific and widespread regional sampling in SEAK before and after SPLASH. The *A8* haplotype differs from the *A−* haplotype by one base pair and, as inferred from our pedigree reconstruction, likely arose through inheritance and segregation of *A−*/*A8* heteroplasmy, operating as an intermediate step between the homoplasmic *A−* and the *A8* haplotypes.

We consider the alternate hypothesis of paternal leakage to be unlikely. If paternal leakage was responsible for the heteroplasmy outlined here, it would be expected to represent a known or existing haplotype in the population; this was not the case. There are various mechanisms preventing paternal leakage from occurring in vertebrate species (Birky 1995). For example, in mammals, the female cell actively tags sperm with ubiquitin, causing proteolytic digestion (Sutovsky 2003). Paternal mtDNA inheritance in most species is further constrained by extreme dilution with maternal mtDNA in the zygote (Wolff and Gemmell 2008).

This is not the first time that mtDNA heteroplasmy has been reported in the humpback whale. Using restriction fragment analysis, Baker et al. (1990) reported heteroplasmy in one individual sampled in Hawaii using the *AccI* restriction enzyme. However, review of the *AccI* cut sites showed this is not consistent with the C/T transition at position 283 of the A8 haplotype reported in this study. More recently, Baker et al. (2013) reported heteroplasmy in 18 humpback whales from among the 1,805 individuals identified by DNA profiling during the SPLASH project. Although the specific heteroplasmic sites were not investigated at the time, we subsequently determined that 2 of these 18 unspecified heteroplasmic individuals had the *A−*/*A8* heteroplasmic haplotype and were included in the 20 reported here.

### Inheritance and segregation of heteroplasmy

Although we were not able to identify the “founder female” of the *A−*/*A8* heteroplasmy, we were able to demonstrate the likely inheritance and segregation of the heteroplasmic haplotype and the subsequent fixation of the *A8* haplotype by pedigree reconstruction. Similarly, McLeod and White (2009) traced mitochondrial control region heteroplasmy through 3 generations of North Atlantic right whales and identified the complete segregation of the mutant variant. As in our study, the ability to track heteroplasmy through multiple generations of the North Atlantic right whale was attributed to the long-term database of identification records and the large number of observed mother/offspring relationships for individual whales.

### Intergeneration change in mtDNA haplotypes

Through our constructed pedigrees, we demonstrate that first-order relatives can have varying proportions of parental (*A−*) and derived (*A8*) nucleotides, resulting in a variable pattern of heteroplasmy and apparent homoplasmy (the presence of only one mtDNA haplotype within an individual) in a mother and her offspring. Although we documented apparent fixation of *A8* in 2 generations, this was not the case in all pedigrees. In one case, an *A−*/*A8* heteroplasmic mother had an offspring apparently fixed for the parental variant (*A−*). In another, a female with the parental variant (*A−*) had an *A−*/*A8* heteroplasmic offspring. These intergenerational changes in mtDNA haplotypes are likely caused by the fluctuations in the number of mitochondrial organelles per cell during embryogenesis or oogenesis. This fluctuation causes only a subset of the mtDNA to proliferate in successive generations (Bergstrom and Pritchard 1998, White et al. 2008).

Tracking of heteroplasmy through a maternal lineage is challenging as inheritance of heteroplasmy can fluctuate depending on which cells, and at which developmental stage, the mutation originated. Detecting point-mutation heteroplasmy also depends on the threshold at which we define detection. It is likely that additional, seemingly homoplasmic, individuals have low levels of heteroplasmy that were not detected through conventional sequencing of mtDNA from skin cells. For example, McLeod and White (2009) report an apparently homoplasmic female giving birth to 2 heteroplasmic offspring. Although this could represent parallel mutation, the authors concluded it was more likely that the mother was heteroplasmic but below the detection threshold. Next-generation sequencing offers an opportunity to improve detection and quantification of heteroplasmy in studies of cetacean mtDNA (e.g. Alexander et al. 2013, Thompson et al. 2014).

### Regional fidelity and rise of a new maternal lineage

The *A−/A8* heteroplasmic individuals showed a pattern of distribution and migratory return consistent with strong maternal fidelity. All but one of the individuals was encountered in either SEAK or Hawaii. As Hawaii is the main breeding ground for humpback whales feeding in SEAK (Baker et al. 1986), it is likely that the 4 *A−*/*A8* individuals encountered in Hawaii also migrated to SEAK but were not included in our sample. The one exception to this exclusive fidelity, was the individual sampled in the NGOA. Although infrequent, movement between feeding regions has been reported previously based on photo-identification (Calambokidis et al. 2008) and DNA profiling (Baker et al. 2013).

The observed heteroplasmy and documentation of a derived mtDNA haplotype provide a unique baseline to document change in the frequency of maternal lineages in a recovering population. In McLeod and White (2009), the 2 individuals that became fixed for the mutant mtDNA variant were both male and could not contribute the new haplotype to future generations. In our study, both individuals with the newly arising *A8* haplotype are female. Furthermore, at least 11 of the 20 heteroplasmic individuals were female. Thus, we anticipate that the *A8* haplotype will become more frequent in SEAK over time. We can assess local recruitment of this lineage to SEAK through continued genetic monitoring and photo-identification of offspring of the heteroplasmic and *A8* individuals (Pierszalowski et al. 2016). An increase in frequency of the *A8* haplotype in SEAK will reflect reproductive success and local fidelity of the 2 existing *A8* individuals as well as any new *A8* females that might arise through a parallel fixation of the *A−*/*A8* heteroplasmy. As regional photo-identification and biopsy sampling are ongoing for humpback whales in SEAK and other regions of Alaska (e.g. Gabriele et al. 2022), it is also possible to relate the dispersal of this newly arising maternal lineage to more conventional measures of maternal gene flow.

## Supplementary Material

esac042_suppl_Supplementary_Material

## Data Availability

The sequence for the novel haplotype, *A8*, has been submitted to GenBank [OP185707].
