# Supplementary material for: mtDNA heteroplasmy gives rise to a new maternal lineage in North Pacific humpback whales (Megaptera novaeangliae)
Source: J Hered. 2022 Sep 23;114(1):14–21. doi: 10.1093/jhered/esac042 (PMC10019026; doi:10.1093/jhered/esac042)
Supplement: esac042_suppl_Supplementary_Material [file esac042_suppl_supplementary_material.docx]

Supplementary Material

Pierszalowski SP, Steel DJ, Gabriele CM, Neilson JL, Vanselow PBS, Cedarleaf JA, Straley JM, Baker CS. mtDNA heteroplasmy gives rise to a new maternal lineage in North Pacific humpback whales (*Megaptera novaeangliae*)

*Sup Mat Table 1*. The 20 individual North Pacific humpback whales found to be heteroplasmic for *A-*/*A8* haplotypes and their corresponding identification codes for the SEAK Regional Database and the SPLASH Program. Also listed, in bold, are the two individuals with the novel *A8* haplotype. *This individual was identified with a temporary SEAK photo code, UASE-JMS-20031009-1-131.

| **SEAK ID / SPLASH ID** | **Location of Tissue Sample** | **Haplotype** | **Sex** |
| --- | --- | --- | --- |
| 193 / 474448 | SEAK | *A-/A8* | F |
| 196 / n.a. | SEAK | *A-/A8* | F |
| 215 / 474268 | SEAK | *A-/A8* | F |
| 875 / 470307 | SEAK | *A-/A8* | M |
| 1336 / 474397 | SEAK | *A-/A8* | F |
| 1345 / n.a. | SEAK | *A-/A8* | F |
| 1756 / 574115 | SEAK | *A-/A8* | n.a. |
| 1795 / 574356 | SEAK | *A-/A8* | n.a. |
| 1809 / 474335 | SEAK | *A-/A8* | M |
| 1817 / 470535 | SEAK | *A-/A8* | M |
| 2002 / 474117 | SEAK | *A-/A8* | M |
| 2062 / 474108 | SEAK | *A-/A8* | F |
| 2119 / 700630 | SEAK | *A-/A8* | F |
| n.a. / 700572 | SEAK | *A-/A8* | F |
| n.a.* / n.a. | SEAK | *A-/A8* | n.a. |
| n.a. / 430048 | HI | *A-/A8* | F |
| n.a. / 430697 | HI | *A-/A8* | M |
| n.a. / 430294 | HI | *A-/A8* | F |
| n.a. / 700227 | HI | *A-/A8* | F |
| n.a. / 472647 | NGOA | *A-/A8* | M |
| **1812 / 474422** | **SEAK** | ***A8*** | **F** |
| **2328 / n.a.** | **SEAK** | ***A8*** | **F** |

|  | **Microsatellite loci as described in Baker et al. (2013)** | | | | | | | | | | | | | | | | | | | |
| --- | --- | --- | --- | --- | --- | --- | --- | --- | --- | --- | --- | --- | --- | --- | --- | --- | --- | --- | --- | --- |
| **SEAK ID / SPLASH ID** | **Ev14a** | **Ev14b** | **Ev37a** | **Ev37b** | **Ev96a** | **Ev96b** | **GATA28a** | **GATA28b** | **GATA417a** | **GATA417b** | **GT211a** | **GT211b** | **GT23a** | **GT23b** | **GT575a** | **GT575b** | **rw4-10a** | **rw4-10b** | **rw48a** | **rw48b** |
| 193 / 474448 | 131 | 133 | 208 | 212 | 159 | 161 | 147 | 147 | 199 | 226 | 104 | 116 | 115 | 121 | 0 | 0 | 198 | 204 | 116 | 116 |
| 196 / n.a. | 0 | 0 | 212 | 216 | 159 | 163 | 147 | 147 | 199 | 214 | 104 | 116 | 115 | 115 | 163 | 163 | 198 | 206 | 116 | 116 |
| 215 / 474268 | 131 | 137 | 198 | 208 | 159 | 163 | 147 | 147 | 207 | 214 | 106 | 110 | 111 | 115 | 163 | 163 | 196 | 196 | 112 | 116 |
| 875 / 470307 | 131 | 139 | 198 | 208 | 157 | 163 | 147 | 147 | 214 | 226 | 104 | 106 | 111 | 115 | 153 | 163 | 198 | 204 | 116 | 116 |
| 1336 / 474397 | 131 | 131 | 208 | 208 | 157 | 161 | 147 | 147 | 226 | 226 | 104 | 112 | 115 | 117 | 153 | 153 | 196 | 204 | 116 | 116 |
| 1345 / n.a. | 131 | 131 | 208 | 212 | 161 | 163 | 147 | 155 | 195 | 199 | 104 | 116 | 111 | 115 | 149 | 163 | 196 | 204 | 116 | 116 |
| 1795 / 574356 | 131 | 133 | 200 | 202 | 161 | 163 | 147 | 147 | 206 | 210 | 110 | 112 | 111 | 113 | 147 | 153 | 196 | 196 | 116 | 118 |
| 1809 / 474335 | 131 | 133 | 200 | 200 | 161 | 161 | 147 | 147 | 0 | 0 | 106 | 116 | 113 | 115 | 0 | 0 | 196 | 198 | 112 | 116 |
| 2002 / 474117 | 131 | 131 | 198 | 200 | 163 | 163 | 0 | 0 | 207 | 214 | 106 | 106 | 113 | 115 | 163 | 163 | 196 | 196 | 116 | 116 |
| 2119 / 700630 | 131 | 133 | 204 | 208 | 157 | 159 | 147 | 147 | 214 | 226 | 104 | 106 | 111 | 115 | 153 | 153 | 196 | 196 | 116 | 118 |
| n.a. / 700572 | 131 | 133 | 192 | 198 | 159 | 165 | 155 | 159 | 195 | 207 | 106 | 116 | 111 | 115 | 151 | 163 | 196 | 196 | 118 | 118 |
| n.a. / 430048 | 131 | 133 | 192 | 198 | 159 | 163 | 155 | 155 | 214 | 226 | 106 | 116 | 111 | 115 | 153 | 153 | 196 | 204 | 116 | 116 |
| n.a. / 430697 | 131 | 131 | 198 | 210 | 157 | 163 | 147 | 183 | 218 | 222 | 106 | 110 | 0 | 0 | 0 | 0 | 196 | 196 | 112 | 116 |
| n.a. / 430294 | 133 | 139 | 198 | 198 | 161 | 163 | 147 | 179 | 226 | 226 | 116 | 116 | 119 | 119 | 151 | 151 | 196 | 204 | 112 | 120 |
| n.a. / 700227 | 131 | 131 | 192 | 198 | 163 | 163 | 147 | 147 | 191 | 214 | 106 | 110 | 113 | 119 | 153 | 155 | 196 | 198 | 112 | 116 |
| n.a. / 472647 | 131 | 135 | 192 | 206 | 161 | 161 | 147 | 155 | 195 | 207 | 104 | 106 | 111 | 115 | 153 | 153 | 196 | 196 | 112 | 112 |
| **1812 / 474422** | **131** | **131** | **198** | **216** | **159** | **161** | **147** | **147** | **199** | **206** | **110** | **116** | **111** | **115** | **157** | **163** | **196** | **206** | **116** | **116** |
| **2328 / n.a.** | **131** | **133** | **214** | **216** | **159** | **161** | **147** | **155** | **199** | **206** | **116** | **116** | **111** | **115** | **151** | **157** | **196** | **196** | **116** | **116** |

*Sup Mat Table 2*. Microsatellite genotypes for 16 of the 20 individual humpback whales found to be heteroplasmic for *A-*/*A8* haplotypes and two individuals with the novel *A8* haplotype (in bold). DNA quality or quantity was insufficient for genotyping of 4 individuals listed in Sup Mat Table 1.
